# Supplementary material for: Comprehensive Analysis of Ubiquitome Changes in Nicotiana benthamiana after Rice Stripe Virus Infection
Source: Viruses. 2022 Oct 26;14(11):2349. doi: 10.3390/v14112349 (PMC9694600; doi:10.3390/v14112349)
Supplement: Supplementary file 1 [file viruses-14-02349-s001.zip › viruses-1958738-supplementary materials excluing Table S2.pdf]

## Supplemental Materials:

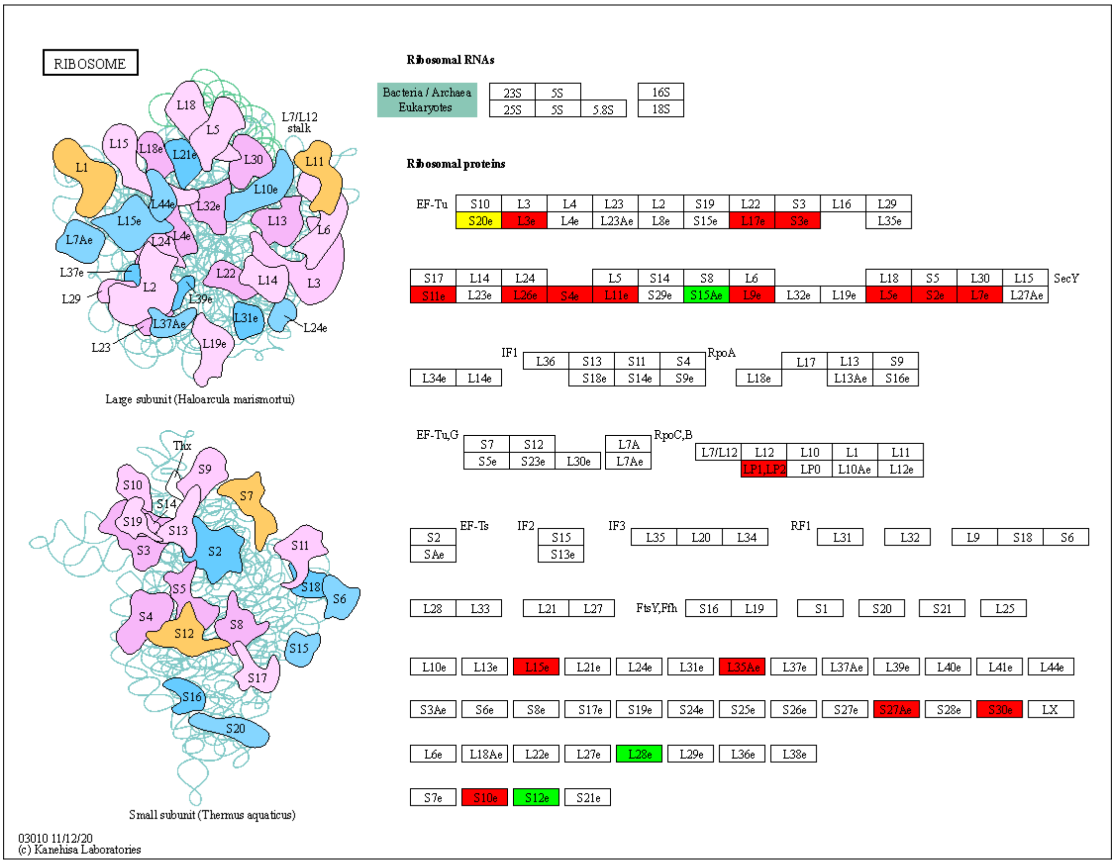


**Figure S1.** Proteins with up regulated Kub sites were significantly enriched in the ribosome pathway. Red square frame means the protein possesses up regulated Kub site. Green square frame means the protein possesses down regulated Kub site. Yellow square frame means the protein both have up- and down-regulated Kub sites.

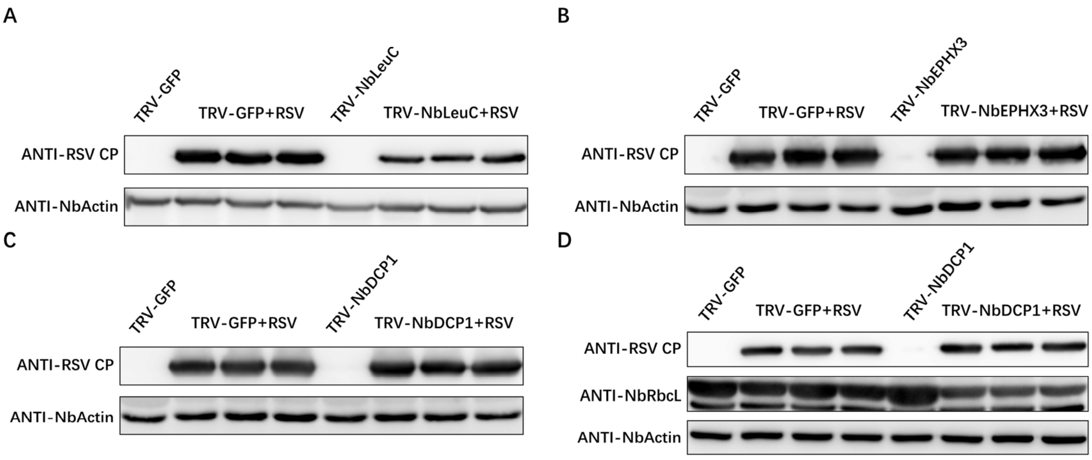


**Figure S2.** Western blotting analysis of RSV CP accumulation. The RSV CP accumulation comparison between *N. benthamiana* plants pre-inoculated with (A) TRV-GFP (control) and TRV-NbLeuC or (B) TRV-GFP (control) and TRV-NbEPHX3 or (C) TRV-GFP (control) and TRV-NbDCP1 at 15 dpi. NbActin was used as a loading control. (D) The RSV CP and NbRbcL accumulation comparison between *N. benthamiana* plants pre-inoculated with TRV-GFP (control) and TRV-NbDCP1 at 30 dpi. NbActin was used as a loading control.

**Table S1.** Primes and genes used for plasmid construction and RT-qPCR.

| | **Primer** | **Sequence** | **Annealing temperature** | Amplification product (accession number) |
|---|---|---|---|---|
| Expression vector | RSV NS2-F | CGATAGGGATCCTGGCTCGAG ATGGCATTACTCCTTTTCAATG | 54°C | RSV NS2 CDS (GenBank: DQ333943.1 81-680 nt) |
| | RSV NS2-R | GTGGTCCTTATAGTCGAGCTCC ATTAGAATAGGGCACTCATGT | | |
| | RSV NS3-F | CGATAGGGATCCTGGCTCGAG ATGAACGTGTTCACATCGTC | 55°C | RSV NS3 CDS (GenBank: DQ333944.1 66-701 nt) |
| | RSV NS3-R | GTGGTCCTTATAGTCGAGCTCC AGCACAGCTGGAGAGCTG | | |
| | RSV CP-F | CGATAGGGATCCTGGCTCGAG ATGGGCACCAACAAGCCAG | 59°C | RSV CP CDS (GenBank: DQ333944.1 1416-2384 nt) |
| | RSV CP-R | GTGGTCCTTATAGTCGAGCTCG TCATCTGCACCTTCTGCCT | | |
| VIGS vector | VIGS NbLeuC-F | TCTAGACCCCCCATATCTTCCTT CTGT | 57°C | NbLeuC segment (Sol genomics: Niben101Scf00859g01014) |
| | VIGS NbLeuC-R | CTCGAGAGCAGCAGCAGAAG AGGAAG | | |
| | VIGS NbEPHX3-F | TCTAGAGTATTTCAGCTTGTTTC GTCC | 55°C | NbEPHX3 segment (Sol genomics: Niben101Scf10737g00028) |
| | VIGS NbEPHX3-R | CTCGAGATCCATGAGGGTAAT GTTGCTG | | |
| | VIGS NbDCP1-F | TCTAGAATCTGGAGCAATCCTA GGGT | 58°C | NbDCP1 segment (Sol genomics: Niben101Scf01750g14030) |
| | VIGS NbDCP1-R | CTCGAGGCGGTTGATAACTCCT GCAAC | | |
| RT-qPCR | QPCR NbLeuC-F | CACTTGCACAGTGCGGTTC | 60°C | NbLeuC segment (Sol genomics: Niben101Scf00859g01014) |
| | QPCR NbLeuC-R | TCTTCCCGCAATTAAGCAGCTC G | | |
| | QPCR NbEPHX3-F | GGCCTTAGCTAAGTCACCAGC | 60°C | NbEPHX3 segment (Sol genomics: Niben101Scf10737g00028) |
| | QPCR NbEPHX3-R | CGTCCAGATCACCCACGAC | | |
| | QPCR NbDCP1-F | TACAGTTCCAGGGCAGCCAT | 60°C | NbDCP1 segment (Sol genomics: Niben101Scf01750g14030) |
| | QPCR NbDCP1-R | GATGCATCAAGGAGGGGCCT | | |
| | QPCR NbActin-F | AAGCTGCAGGTATCCATGAGA CTA | 60°C | NbActin segment (Sol genomics: Niben101Scf06954g00015) |
| | QPCR NbActin-R | CAATCCAGACACTGTACTTTCT CTC | | |

**Table S3.** Detailed information of the ubiquitination motifs.

| Motif | Motif Score | Foreground | | Background | | Fold Increase |
|---|---|---|---|---|---|---|
| | | Matches | Size | Matches | Size | |
| ..........KG......... | 16.00 | 366 | 2508 | 52460 | 904646 | 2.5 |
| ........AKK.......... | 23.65 | 48 | 2142 | 4152 | 852186 | 4.6 |
| .........GK.......... | 16.00 | 310 | 2094 | 55079 | 848034 | 2.3 |
| .........RK.......... | 16.00 | 307 | 1784 | 52308 | 792955 | 2.6 |
| ..E......KK.......... | 24.13 | 55 | 1477 | 5483 | 740647 | 5.0 |
| .........KK.......... | 16.00 | 235 | 1422 | 60514 | 735164 | 2.0 |
| .........SK.......... | 16.00 | 221 | 1187 | 69198 | 674650 | 1.8 |
| ..........K.G........ | 9.82 | 110 | 966 | 36260 | 605452 | 1.9 |
| .........AK.......... | 9.23 | 131 | 856 | 50188 | 569192 | 1.7 |
| ..........K.A........ | 6.03 | 83 | 725 | 34182 | 519004 | 1.7 |
| .......E..K.......... | 6.03 | 96 | 642 | 43864 | 484822 | 1.7 |
